# E-GuARD: expert-guided augmentation for the robust detection of compounds interfering with biological assays

**DOI:** 10.1186/s13321-025-01014-3

**Published:** 2025-04-29

**Authors:** Vincenzo Palmacci, Yasmine Nahal, Matthias Welsch, Ola Engkvist, Samuel Kaski, Johannes Kirchmair

**Affiliations:** 1 Department of Pharmaceutical Sciences, Division of Pharmaceutical Chemistry, Faculty of Life Sciences, University of Vienna, 1090 Vienna, Austria; 2 Vienna Doctoral School of Pharmaceutical, Nutritional and Sport Sciences (PhaNuSpo), University of Vienna, 1090 Vienna, Austria; 3 Department of Computer Science, Aalto University, Espoo, Finland; 4 Molecular AI, Discovery Sciences, BioPharmaceuticals R&D, AstraZeneca, Gothenburg, Sweden; 5 Christian Doppler Laboratory for Molecular Informatics in the Biosciences, Department for Pharmaceutical Sciences, University of Vienna, 1090 Vienna, Austria; 6 Department of Computer Science and Engineering, Chalmers University of Technology, Gothenburg, Sweden; 7 Department of Computer Science, University of Manchester, Manchester, UK

## Abstract

**Abstract:**

Assay interference caused by small organic compounds continues to pose formidable challenges to early drug discovery. Various computational methods have been developed to identify compounds likely to cause assay interference. However, due to the scarcity of data available for model development, the predictive accuracy and applicability of these approaches are limited. In this work, we present E-GuARD, a novel framework seeking to address data scarcity and imbalance by integrating self-distillation, active learning, and expert-guided molecular generation. E-GuARD iteratively enriches the training data with interference-relevant molecules, resulting in quantitative structure-interference relationship (QSIR) models with superior performance. We demonstrate the utility of E-GuARD with the examples of four high-quality data sets on thiol reactivity, redox reactivity, nanoluciferase inhibition, and firefly luciferase inhibition. Our models reached MCC values of up to 0.47 for these data sets, with two-fold or higher improvements in enrichment factors compared to models trained without E-GuARD data augmentation. These results highlight the potential of E-GuARD as a scalable solution to mitigating assay interference in early drug discovery.

**Scientific contribution:**

We present E-GuARD, an innovative framework that combines iterative self-distillation with guided molecular augmentation to enhance the predictive performance of QSAR models. By allowing models to learn from newly generated, informative compounds through iterations, E-GuARD facilitates the understanding of underrepresented structural patterns and improves performance on unseen data. When applied across different interference mechanisms, E-GuARD consistently outperformed standard approaches. E-GuARD establishes the foundation for further research into dynamic data enrichment and more robust molecular modeling.

**Supplementary Information:**

The online version contains supplementary material available at 10.1186/s13321-025-01014-3.

## Introduction

High-throughput screening (HTS) is of fundamental importance to modern drug discovery, allowing for the rapid assessment of hundreds of thousands of compounds for activity on biomacromolecular targets of interest [1]. However, a substantial number of hits reported by HTS technologies may be linked to assay interference caused by compound aggregation, direct interference with the detection methods, or nonspecific chemical reactions with assay components [2, 3]. Assay interfering compounds are often called “bad actors” or “nuisance compounds” [4], and are common to chemical libraries, representing a bottleneck for the early drug development pipeline.

Today, various experimental approaches, such as counter-screenings or orthogonal assays, are routinely employed to identify assay-interfering compounds and false-positive assay readouts [2, 5]. While these experimental methods are essential in drug and probe discovery, they are substantial in cost and cannot always be applied retrospectively during chemical library screening and preparation, particularly when working with large libraries. On the other hand, computational methods have emerged as a promising alternative for predicting assay interference [2] as they offer a complementary strategy by identifying potential assay interference patterns earlier in the discovery process, which may help prioritize compounds for experimental follow-up and optimize screening resources.

Among the most relevant models are several machine learning approaches, including HitDexter 3.0 [5, 6], which predicts compounds likely to show frequent hitter behavior, and PISA-T [7], which flags compounds likely to interfere with fluorescence-based assays. These models leverage extensive HTS data sets but are agnostic of interference mechanisms.

Considering the mechanisms underlying assay interference phenomena can improve the accuracy and relevance of predictions. Therefore, researchers have explored strategies for training machine learning approaches on experimental assay interference data obtained, e.g., via counter-screens and orthogonal assays [8–10]. However, data scarcity and class imbalance prove challenging for model development.

To expand the availability of measured data for model development, Alves et al. [11] selected compounds from the NPACT data set and subsequently tested them in-house using HTS assays. This ensured all experimental data were generated under consistent conditions, minimizing variation across assays. Their data collection is among the most comprehensive in the field and includes molecular structures associated with measured data on thiol reactivity (TR), redox reactivity (RR), nanoluciferase inhibition (NI), and firefly luciferase inhibition (FI). Each of the four interference classes is represented by approximately 5,000 measured compounds, with interference rates ranging from 1.5% to 20%, depending on the data set. The authors demonstrated that their data collection is suitable for training reliable machine learning models for assay interference prediction. This resulted in the development of the “Liability Predictor”, an online tool featuring XGBoost-based quantitative structure-interference (QSIR) models that accurately identify interfering compounds.

Although the compiled data sets are of great value to research, challenges related to data scarcity and class imbalance remain. Theoretical approaches to address class imbalance range from basic techniques, such as over-sampling the minority class or under-sampling the majority class [12], to more advanced methods, such as weighted loss functions for hill-climbing algorithms [13]. Data augmentation is a further, effective strategy to alleviate class imbalance and data scarcity by generating synthetic examples to enrich the training set [14, 15]. For example, techniques such as SMOTE [16] (Synthetic Minority Over-sampling Technique) are widely utilized in cheminformatics for their flexibility and straightforward implementation. More recent applications of data augmentation include the introduction of noisy labels via self-distillation, which is the process of first training a “teacher” model on labeled data and then using its predictions to train a “student” model with the same architecture [17]. Self-distillation has been empirically observed to provide model performance gains on various tasks, including image recognition [18] and protein structure prediction [19]. Building on this concept, Liu et al. [20] developed the Pseudo Label Augmented Neural System (PLANS) for quantitative structure–activity relation (QSAR) modeling applications. PLANS uses a teacher model trained on fully labeled data to generate pseudo-labels for a large pool of unlabeled compounds collected from the ChEMBL database. This self-distillation approach enhanced predictive performance when tested on cytochrome P450 substrate prediction and Tox21 data sets [21]. However, as stated by the authors, PLANS introduces a significant amount of noise, likely due to the introduction of model-generated labels when using the complete ChEMBL database as a source of unlabeled data. This noise can confuse the model, resulting in a notable decline in performance.

To overcome the limitations and challenges of existing approaches, in this work, we combine self-distillation with tailored molecular generation and active learning [22, 23] in a new framework that we call E-GuARD (Expert-Guided Augmentation for the Robust Detection of Compounds Interfering with Biological Assays).

**Fig. 1:**
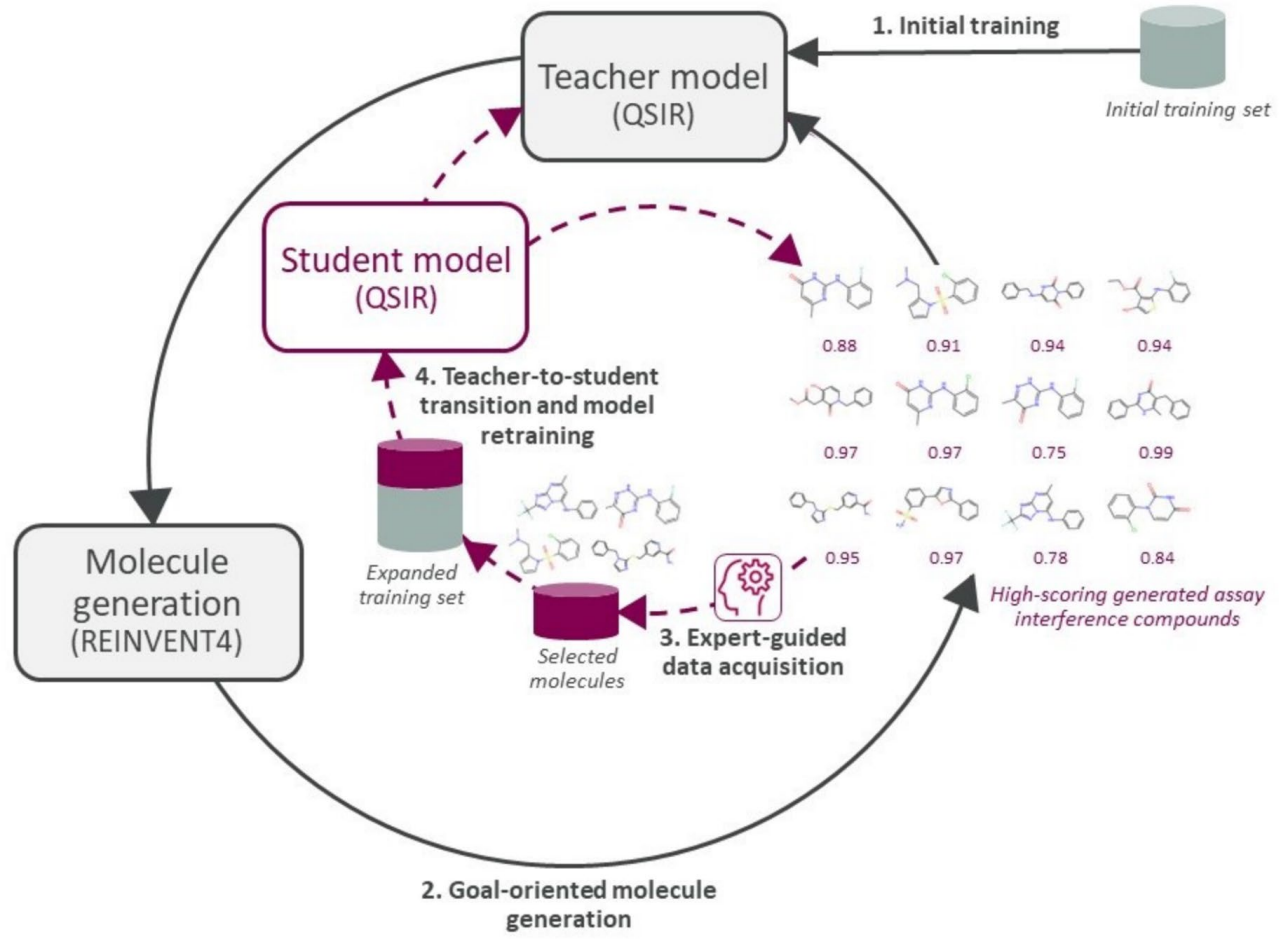
Overview of the E-GuARD workflow, which involves the iterative process of molecular generation, expert-guided data augmentation, and self-distillation. First, a teacher model is trained. The teacher model is then used to guide molecule generation towards interfering compounds (outer loop represented by black arrows). Once a pre-defined number of outer loop iterations has been completed, the teacher model becomes the student model and is iteratively updated through expert-guided data augmentation and self-distillation (inner loop represented by dashed red arrows)

E-GuARD (Fig. 1) builds upon the concept of self-distillation, with two key distinctions: (i) instead of sourcing unlabeled data from existing data sets (e.g., the ChEMBL database), E-GuARD generates new chemical structures with the de novo molecular design tool REINVENT4 [24]; (ii) E-GuARD adds unlabeled data to the training set following expert guidance emulated with MolSkill [25]. This approach is designed to balance exploration of the chemical space with targeted refinement, leveraging both de novo molecule generation and expert-guided feedback to optimize the discovery process.

Specifically, E-GuARD starts by enhancing an initial, small training set by iteratively adding selected compounds from a pool of molecules generated with REINVENT4. The teacher model guides the algorithm toward relevant regions of the chemical space. The loop is executed for a defined number of iterations (in our study, five iterations), with new molecules selected using one of five acquisition functions. At the end of each iteration, the teacher model transitions into the student role and is retrained on the augmented training data set, enabling continuous refinement and improvement for each subsequent iteration. To minimize noise and ensure that compounds considered are relevant to drug discovery, a method to proxy human feedback is included in the optimization cycle. The proxy human feedback is generated with MolSkill, a neural network model developed to emulate the decision-making process of medicinal chemists. MolSkill’s feedback is used in combination with two acquisition functions to select molecules to be added to the training set. This component indirectly injects human expertise into the reinforcement learning loop of REINVENT4, resulting in the generation of drug-like molecules based on diverse scaffolds.

We explored E-GuARD’s ability to improve the prediction of four mechanisms of assay interference: TR, RR, NI, and FI. For each interference mechanism, we performed ten independent runs of iterative self-distillation. Compared to baseline QSIR models, the QSIR models generated with the E-GuARD approach showed improved predictive performance across both internal and external test sets.

Analyzing the student model's evolution, we show that E-GuARD improves the detection of compounds interfering with biological assays, by enabling the learning of new features across iterations. Additionally, evaluating the QED scores and diversity of the generated compounds shows that the newly added compounds remain diverse and relevant to drug discovery. This demonstrates that the observed performance improvements stem from learning novel features.

## Materials and methods

### Data collection

Sets of measured data on the interference of 5,098 compounds with biological assays via TR, RR, NI, and FI were obtained from Alves et al. [11]. For each data set, 25% of the compounds were randomly selected and assigned to a test set. The remaining 75% of compounds were divided into five subsets of equal size for cross-validation and hyperparameter optimization (Table 1). All splits preserved the class distribution of the initial data set.

**Table 1:** Overview of the data sets employed in this work

Type of interference	Training set		Test set	
	# positive compounds	# negative compounds	# positive compounds	# negative compounds
Thiol reactivity (TR)	811	3035	198	764
Redox reactivity (RR)	113	3781	29	945
Firefly luciferase inhibition (FI)	90	3834	28	953
Nanoluciferase inhibition (NI)	82	3836	15	965

To evaluate the impact of E-GuARD on QSAR model performance more rigorously, we conducted an external validation using a dataset derived from PubChem for firefly luciferase interference (AID411), which was previously employed in the Luciferase Advisor study [26]. SMILES and corresponding interference labels were obtained from PubChem, and to exclude any data leakage, compounds overlapping with the training set were identified by converting molecules to Morgan3 fingerprints and conducting an exact match search. This resulted in the removal of 24 molecules. The final external dataset comprised 70,619 unique compounds, including 1571 interfering and 69,048 non-interfering compounds, none of which were utilized during model training.

### E-GuARD workflow

E-GuARD utilizes a teacher-student loop to enhance the prediction of interfering compounds. This loop, illustrated in Fig. 1, consists of four key steps:

1. *Initial Training of the Teacher Model* A QSIR model is initially trained on the available training data set.
2. *Goal-Oriented Molecule Generation* New molecules are generated and scored using the teacher model.
3. *Expert-Guided Data Acquisition* Compounds are selected using one of five acquisition functions, including expert-based scoring with MolSkill.
4. *Teacher-to-Student Transition and Model Retraining* The training set is augmented with the selected compounds, and the student model is retrained. The student model then becomes the teacher for the next iteration.

The following sections explain the details underlying the four steps and the tools utilized.

#### Teacher-student model for interference prediction (QSIR)

The balanced random forest (BRF) classifier algorithm, implemented in the imbalanced-learn Python library [27], was chosen as the QSIR teacher model to provide a baseline consistent with the “Liability predictor” [11] performances (see Table S1). The BRF classifier addresses class imbalance by creating bootstrapped subsets with equal representation of each class, thereby mitigating the dominance of the majority class.

A dedicated BRF model was trained for each data set, with hyperparameters (n_estimators, max_depth, min_samples_split, and max_features) optimized using Optuna [28] over 50 trials throughout a fivefold cross-validation procedure. As the input of the machine learning models, the molecular representation of choice was Morgan 3 fingerprints, with a bit length of 2048 bits, generated using the RDKit [29].

The BRF classifiers are then retrained on the augmented training set using the same hyperparameter set determined during the initial optimization.

#### Goal-oriented molecule generation with REINVENT4

The training set is augmented with new molecules generated with REINVENT4. REINVENT4 uses a reinforcement learning framework to optimize molecular generation based on a custom scoring function. In this study, the scoring function for the RL agent feedback, $f(x)$, was defined as Eq. 1,

1 $$f\left(x\right)={\omega }_{1}m\left(x\right)+{\omega }_{2}{wt}\left(x\right)$$

where $m(x)$ represents the interference score computed as the predicted probability of the compound $x$ to be an interfering compound according to the QSIR model, and $wt(x)$ is a molecular weight score designed to prioritize compounds typically relevant to small-molecule drug discovery (160–480 Da) [30]. The weights ${\omega }_{1}$ and ${\omega }_{2}$ were set to 0.8 and 0.2 respectively, with normalization applied.

During each generation step, the REINVENT4 agent executes 250 iterations of optimization, generating 100 compounds per iteration. By the end of the optimization process, a total of 25,000 compounds have been generated. This molecule pool is then filtered using one of five acquisition functions (see the next section for a detailed description) to select the 250 most informative compounds. These compounds are added to the training set to augment the data and retrain the BRF classifier in subsequent iterations.

#### Active data acquisition

Active learning (AL) was employed to iteratively select and add informative compounds to the training set, aiming to increase the likelihood that the added compounds would be diverse and relevant to the modeled task of assay interference prediction.

At the end of each REINVENT4 generation cycle, a pool of compounds ${U}_{r}$ is generated. The generated compounds are obtained by maximizing the reward given by the pre-defined scoring function (Eq. 1). Then, an acquisition criterion is applied to select a subset of compounds from ${U}_{r}$ (Eq. 2) according to

2 $$A\left(x\right)=\alpha {A}_{\text{predictor}}\left(x\right)+\beta {A}_{\text{human}}\left(x\right)$$

where $\alpha {A}_{predictor}(x)$ corresponds to the model evaluation score and $\beta {A}_{human }(x)$ corresponds to the simulated human evaluation score. $\alpha$ and $\beta$ are weighting constants.

First, we employed three different acquisition strategies with $\beta$ set to 0 so that the compound selection from ${U}_{r}$ is based solely on ${A}_{predictor}(x)$:

1. Random Selection: Molecules are randomly selected from ${U}_{r}$.
2. Greedy Selection: The molecules with the highest predicted probabilities of interference are selected from ${U}_{r}$ focusing on the most confident model predictions.
3. Expected Predictive Information Gain (EPIG) Selection [31]: The most informative molecules are selected from ${U}_{r}$ based on their ability to reduce the predictive uncertainty within the top 1000 molecules in ${U}_{r}$. For each $x$ in ${U}_{r}$, EPIG calculates the expected mutual information between the interference labels of $x$ and a randomly sampled $x$* from the target set of top high-scoring 1000 molecules. Mathematically, EPIG is formulated as the expected Kullback–Leibler divergence between the joint distribution $p$*(*$y$*, *$y$* | $x$*, *$x$*) and the product of marginals $p$*(*$y$* |*$x$*)*$p$*(*$y$* | $x$*).

Additionally, the Greedy and EPIG selection strategies were combined with an expert-guided criterion ${A}_{human }(x)$, with both $\alpha$ and $\beta$ set to 1. In this work, ${A}_{human }(x)$ was simulated using the MolSkill score. The following expert-guided acquisition criteria were employed:

4. EPIGSkill: The most informative molecules are selected from ${U}_{r}$ using an integrative scoring system that combines the EPIG score with the expert preference score predicted by MolSkill.
5. GreedySkill: The most informative molecules are selected from ${U}_{r}$ based on an integrative score that combines the Greedy score and expert preference score as predicted by MolSkill.

The various acquisition functions, combined with the simulated expert scoring, steer the generation of compounds toward distinct chemical spaces. This impacts predictor performance and allows the approach to be tailored to specific task requirements.

#### Expert-guided data augmentation with MolSkill

MolSkill is a neural network designed to emulate medicinal chemists' decision-making processes during lead optimization in drug discovery. It applies learning-to-rank techniques to prioritize molecules based on desirability criteria such as drug-likeness and synthetic accessibility. MolSkill is trained on preference feedback from 35 Novartis chemists of varying expertise. They were presented with pairs of drug candidates through a graphical interface and asked to select their preferred option.

In this work, MolSkill was applied as an expert scoring function for data acquisition to identify the most expert-desirable generated molecules to be incorporated into the training set. The inclusion of MolSkill into E-GuARD aims to enhance the model's robustness in detecting challenging, hard-to-detect assay interference compounds that exhibit desirable drug-like properties.

### Evaluation metrics

#### For QSIR model performance

*The Matthews Correlation Coefficient (MCC)* was used as the primary measure of model performance (Eq. 3). The MCC is a balanced metric that takes the true positive (TP), false positive (FP), true negative (TN), and false negative (FN) instances into account:

3 $${\text{MCC = }}\frac{{{\text{TP}} \cdot {\text{TN}} - {\text{FP}} \cdot {\text{FN}}}}{{\sqrt {\left( {\text{TP + FP}} \right)\left( {\text{TP + FN}} \right)\left( {\text{TN + FP}} \right)\left( {\text{TN + FN}} \right)} }}$$

The MCC returns values between − 1 (total disagreement between prediction and observation) and + 1 (perfect agreement).

*The Enrichment Factor (EF)* [32] was employed to evaluate the ability of the model to prioritize true positives, providing a prevalence-adjusted measure of precision (Eq. 4). The EF is a measure of the factor by which a model enriches relevant outcomes compared to random chance:

4 $$\text{EF} = \frac{\text{Precision}}{\frac{\text{TP+FP}}{\text{TP+FP+TN+FN}}}$$

EF values greater than 1 indicate that the model effectively enriches true positives.

#### For the generated compounds

*Imbalance rate (IR)* puts into perspective the proportion of negative and positive examples of the data set, giving a measure for the class imbalance (Eq. 5). IR is computed at each iteration $t$ as:

5 $${\text{IR}}\left(t\right)\text{=}\left|\frac{{\text{Positives}}\left({\text{t}}\right)\text{-Negatives}\left({\text{t}}\right)}{{\text{Positives}}\left({\text{t}}\right)+ \text{Negatives} \left({\text{t}}\right)}\right|$$

*Internal chemical diversity* assesses the chemical diversity within a molecular set *G* (Eq. 6). The metric is limited to [0, 1], and a higher value corresponds to higher diversity in the generated set. Internal chemical diversity is measured as:

6 $${\text{IntDi}}{\text{v}}_{\text{p}}\left(G\right)\text{=}\hspace{0.25em}1\hspace{0.25em}-\hspace{0.25em}\sqrt[p]{\frac{1}{\left|{\text{G}}\right|}{\sum }_{{m}_{1},{m}_{2}\in G}{{\text{Tanimoto}}\left({\text{m}}_{1}\text{,\hspace{0.25em}}{\text{m}}_{2}\right)}^{p}}$$

where $G$ corresponds to the set of generated molecules, represented in this study by their 2048-bit Morgan2 fingerprint vectors. We primarily consider $p=1$ in this work.

The QED score [30] is a quantitative metric of a compound’s drug-likeness. It is based on a combination of physicochemical properties commonly associated with successful drugs, such as reasonable molecular weight and lipophilicity. The QED score ranges from 0 to 1, where higher scores indicate a compound is more likely to have desirable drug-like properties.

*The number of structures matching at least one PAINS alert* is determined using the RDKit Python library through the FilterCatalog. PAINS method. This method employs a set of predefined substructure filters to identify molecules likely to produce false-positive results in HTS assays. The PAINS filter [33] captures structural motifs associated with assay interference, such as reactive groups, or with promiscuous binding properties. While many of these PAINS substructures are associated with redox or thiol reactivity, they can also interfere through other mechanisms, including aggregation and singlet oxygen quenching.

### Model analysis

Centered kernel alignment (CKA) is a method for quantifying the similarity of embedding distributions. Values for CKA range from 0 to 1, where 1 indicates high similarity [34]. Initially developed for NNs, CKA was recently adopted to better capture the similarity between RFs (CKA_rf_) [35], by utilizing a random forest kernel. The random forest kernel compares two data instances based on how often they share a partition in the decision trees of the RF [36]. CKA_rf_ compared with CKA with dot product as the kernel has the advantage that it can capture the inner workings of RFs and is hence utilized in this work to measure the similarity between RFs by comparing the embeddings distributions of the test sets. The implementation of CKA by Abdullah et al. [37] was used to calculate CKA.

## Results

### Characterization of the data sets employed for QSIR model development

This work builds on data compiled for 5,098 compounds measured for TR, RR, NI, and FI [11]. Preprocessing this data according to the protocol outlined in the Methods section removed approximately 200 compounds per data set (Table 1). Ninety-five percent of the remaining molecules have measured data available for all four types of assay interference.

**Fig. 2:**
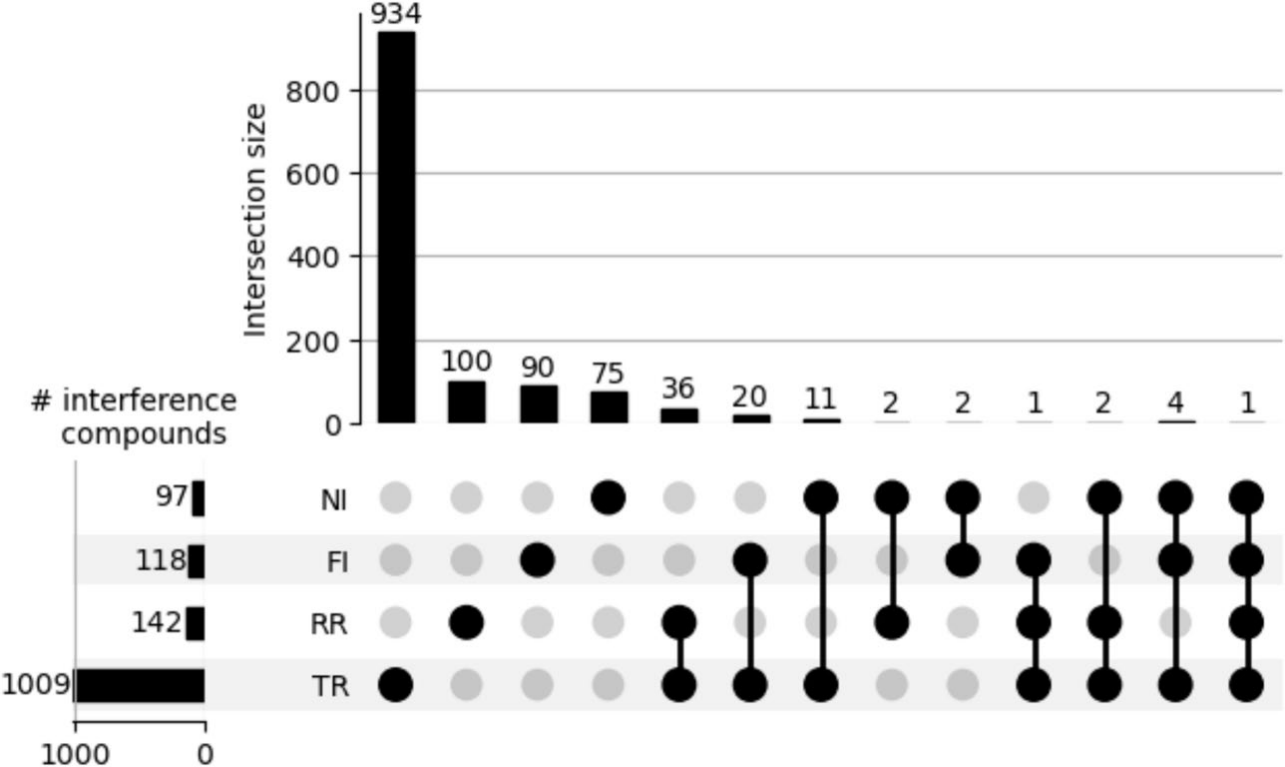
UpSet plot reporting the number of interfering compounds in the individual data sets and their overlaps across the data sets. The horizontal bars indicate each data set’s total number of interference compounds. The vertical bars show the number of interference compounds in the indicated data sets. For example, there are 1009 compounds with confirmed TR and 118 with confirmed FI. Twenty of these compounds show both TR and FI

The UpSet plot in Fig. 2 shows that the overlap between the compounds causing different types of interference is minimal. This indicates that the interference mechanisms are primarily independent from one another. An exception is observed in the RR and TR data sets, where 36 out of 142 compounds involved in redox reactions also exhibit TR. This overlap may also be attributed to the fact that reactions involving thiol moieties can occur through a redox mechanism.

### Training set augmentation via active learning

**Fig. 3:**
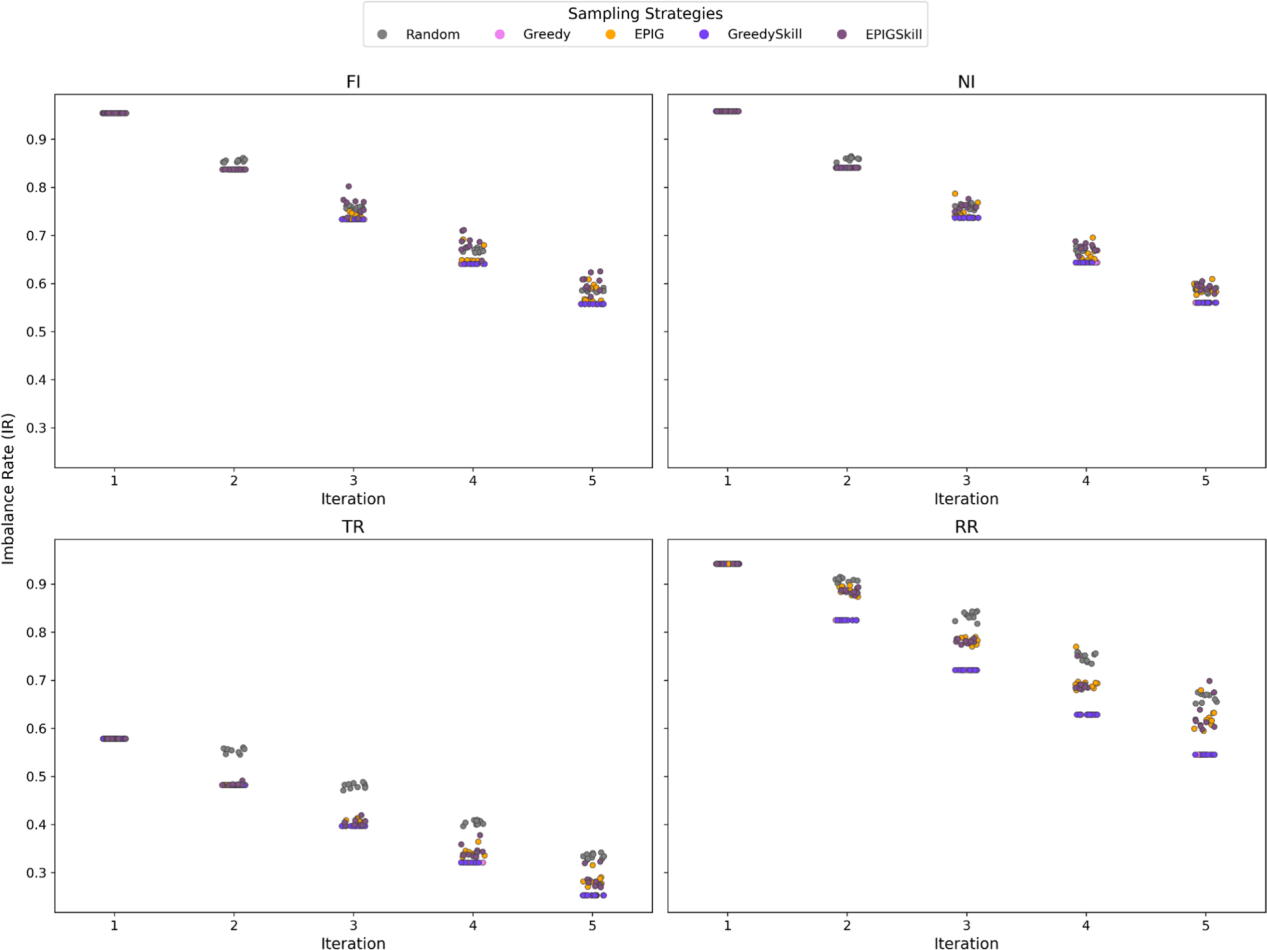
Evolution of the training set IR over five E-GuARD iterations using the five acquisition strategies. Ten repetitions were performed using each acquisition strategy with higher variance among the IR values for Random, EPIG, and EPIGSkill

Subsets of compounds generated through REINVENT4 were selected for inclusion in the training set using five distinct acquisition functions: Random, Greedy, EPIG, GreedySkill, and EPIGSkill (see the Materials and Methods section for detailed descriptions). Figure 3 depicts the augmented training set IR (Eq. 5).

Clearly, the IR decreases for all the data sets during each iteration, highlighting a balancing effect introduced by E-GuARD augmentation. For the most imbalanced data sets (FI, NI, and RR), the IR dropped from 0.97 to 0.60, indicating a progressive balancing effect of the active learning acquisitions. For TR, the effect is less pronounced. Still, at iteration five, E-GuARD enriched the data set with positive samples, achieving almost perfect balancing as indicated by an IR of approximately 0.20.

While all acquisition functions contributed to improving data set balance, the strategies that prioritized the selection of compounds with high interference scores (e.g., Greedy, GreedySkill, and EPIGSkill) consistently added more than 200 likely interfering compounds at each iteration. This result underscores the effectiveness of active learning in mitigating data set imbalance by systematically enriching the training set with relevant and informative samples.

**Fig. 4:**
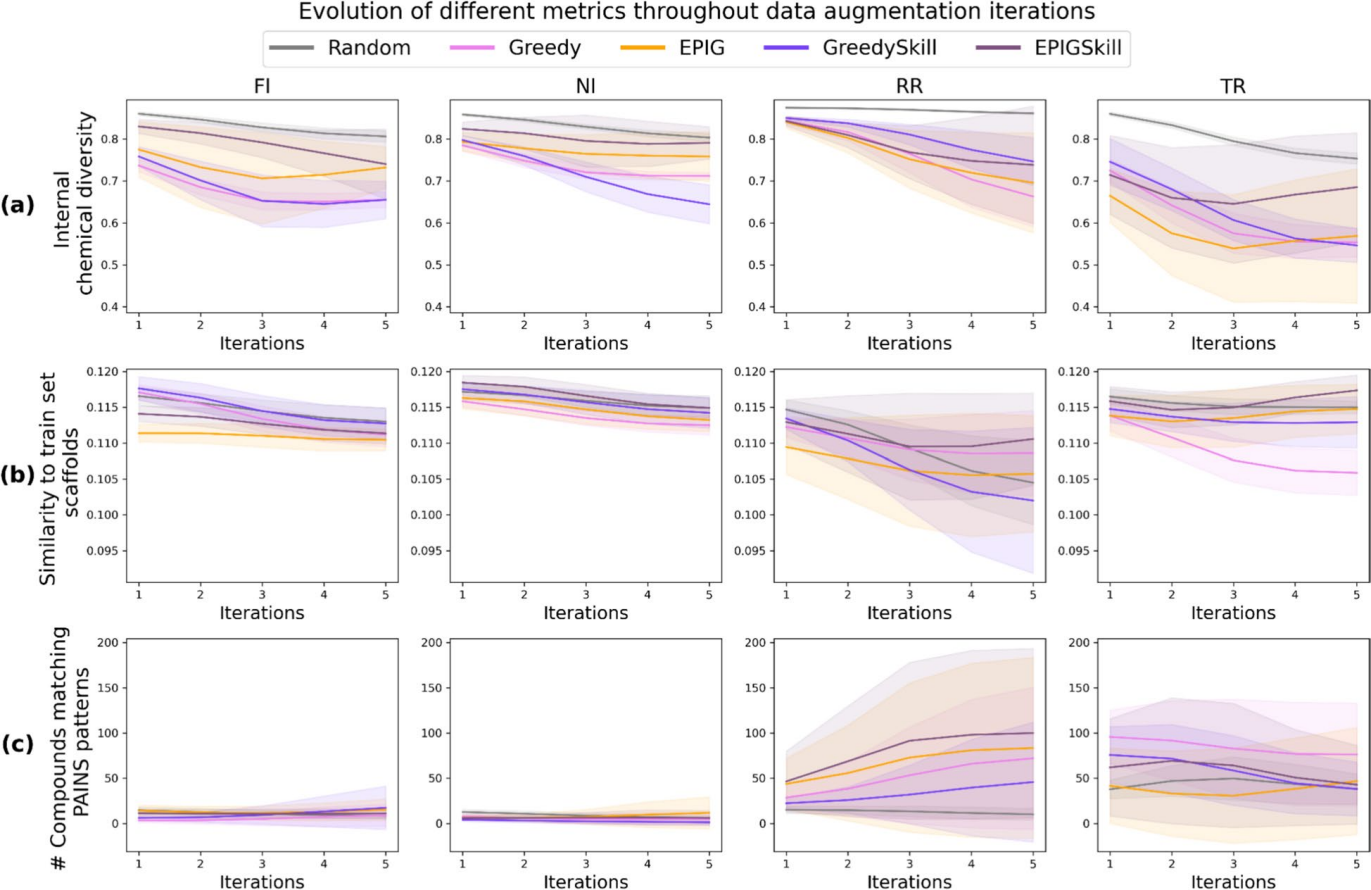
Chemical space analysis of the training set across five E-GuARD iterations: **a** chemical diversity within the generated compounds added to the training set, **b** Tanimoto similarity, computed using Morgan2 fingerprints with 2048 bits, between the newly generated scaffolds and the scaffolds represented in the initial training set, **c** number of compounds matching at least one PAINS pattern

Further, we explored how E-GuARD affects the chemical space of the training set across sampling iterations by analyzing the augmented data set’s internal diversity (Eq. 6), scaffold similarity with the initial training set, and the presence of PAINS substructures (Fig. 4). As shown in Fig. 4a, the internal molecular diversity value decreased, on average, by 20% across the five E-GuARD iterations for every data set. This phenomenon could be attributed to the generative model mode collapse [38], leading to the maximum exploitation of the reward function and resulting in a diminished exploration of new regions of the chemical space. Still, an internal diversity of at least 0.6 was maintained when Random sampling, the Greedy or EPIGSkill acquisition functions were utilized. Random sampling maintained the highest molecular diversity with more than 0.7 Tanimoto distance within the sets of generated molecules. This outcome is expected as REINVENT4 inherently generates diverse samples due to its Diversity Filters functionality. In contrast, uniform sampling from the generated chemical space is performed when no scoring function is applied.

In addition, we computed the scaffold similarity between the initial training sets and newly generated compounds to evaluate the evolution of the explored chemical space. As shown in Fig. 4b, scaffold similarity remained, on average, constant across iterations (values between 0.11 and 0.12), independent of sampling strategies and data sets. These low similarities indicate the novelty of the compounds added to the training set at each iteration, suggesting that E-GuARD successfully explores novel interference-relevant chemical spaces and can potentially expand the model’s possibilities of learning new structures associated with interference.

To further evaluate the impact of E-GuARD on the training chemical space, we analyzed the number of PAINS-containing structures added to the training set at each iteration, as shown in Fig. 4c. The plot reveals that the number of added compounds triggering PAINS alerts for the FI and NI data sets remained below 50 across the five iterations, regardless of the acquisition function used. In contrast, the TR and RR data sets showed enrichment in PAINS-containing compounds from the first iterations, with 50% and 90% of the added compounds containing PAINS substructures for TR and RR data sets, respectively. This was particularly noticeable with the GreedySkill and Greedy acquisition functions. The absence of PAINS in the FI and NI data sets, as well as their notable increase in the RR data set, can be attributed to the nature of PAINS. Most PAINS are associated with redox reactivity and are not linked to mechanisms resulting in luciferase inhibition, explaining their differing distribution across data sets. However, given that PAINS substructures may interfere via alternative mechanisms such as aggregation or singlet oxygen quenching, their presence could contribute to assay-dependent biases beyond redox activity. This suggests that while E-GuARD can enrich data sets with structural patterns associated with different types of assay interference, it may also inadvertently amplify the presence of compounds prone to false positives. The impact of this enrichment depends on the context: on the one hand, it enables the model to learn and recognize interference-prone chemotypes, potentially improving its ability to distinguish true actives from assay artifacts. On the other hand, it could introduce unintended biases if these structures disproportionately influence model predictions and lead to an overrepresentation of PAINS-containing compounds in the acquired data. Therefore, while E-GuARD can be used to shape the training space towards interference compounds, careful interpretation of the generation outcome is necessary to ensure that enrichment does not compromise model generalizability.

**Fig. 5:**
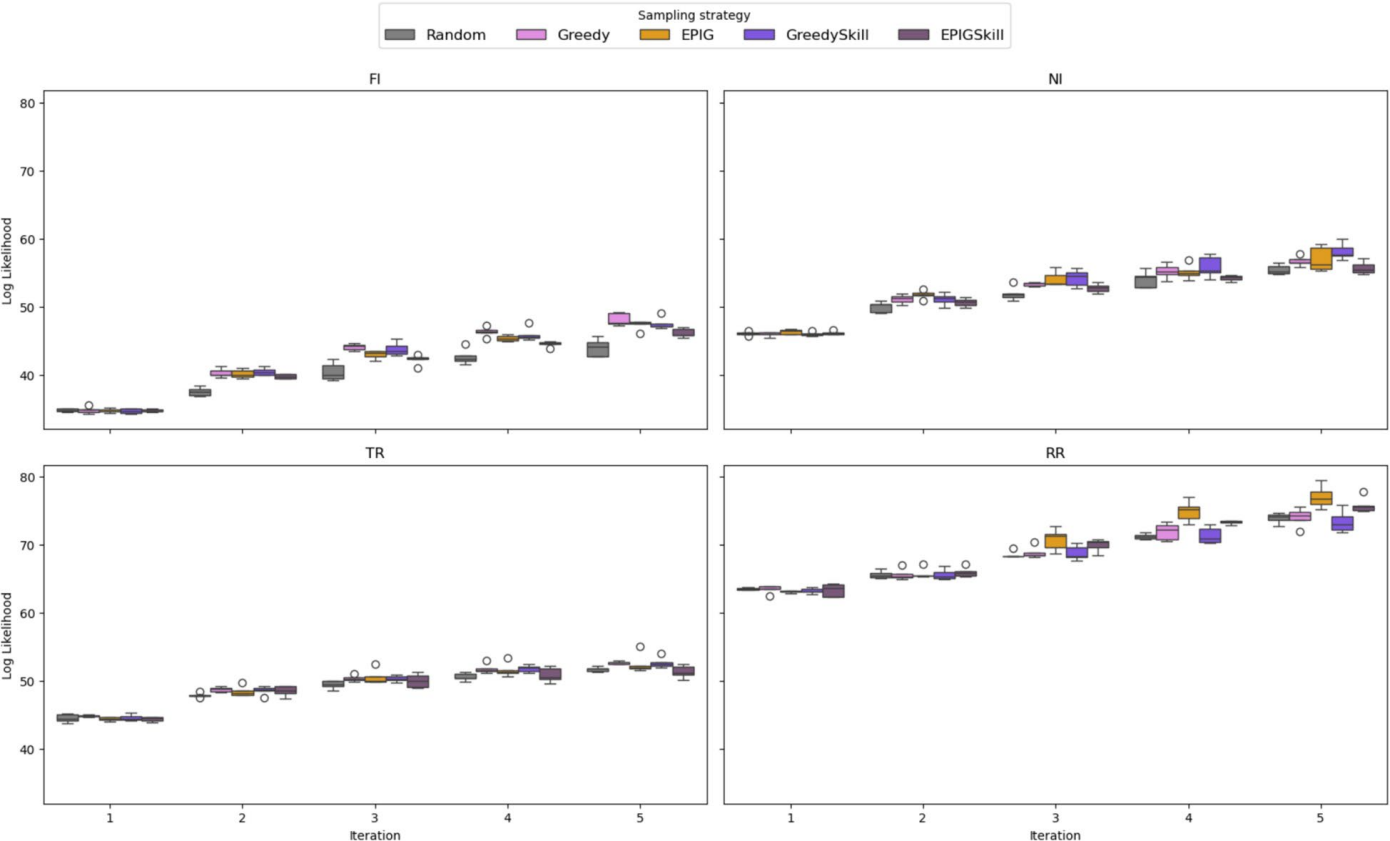
Boxplot reporting the log-likelihood of the putative interfering compounds to be generated by REINVENT4 across iterations for each data set. Each plot compares the likelihood achieved using different acquisition functions (Random, Greedy, EPIG, EPIGSkill, and GreedySkill) throughout five iterations

Additionally, it is essential to assess whether the generative model can produce molecules resembling known interfering compounds. This evaluation helps determine E-GuARD’s ability to enhance the training set with structures relevant to the modeled task. Hence, we analyzed the likelihood of the RL agent to generate the known interfering compounds present in the test set. Figure 5 shows the evolution of the log-likelihood scores computed for known interfering compounds from the test set. Clearly, the boxplot exhibits an upward trend over successive iterations. For all the data sets, the likelihood value increased by at least 50% when the run was completed (i.e., at iteration five), suggesting that the molecule generator learns relevant structures of unseen assay interfering compounds.

**Fig. 6:**
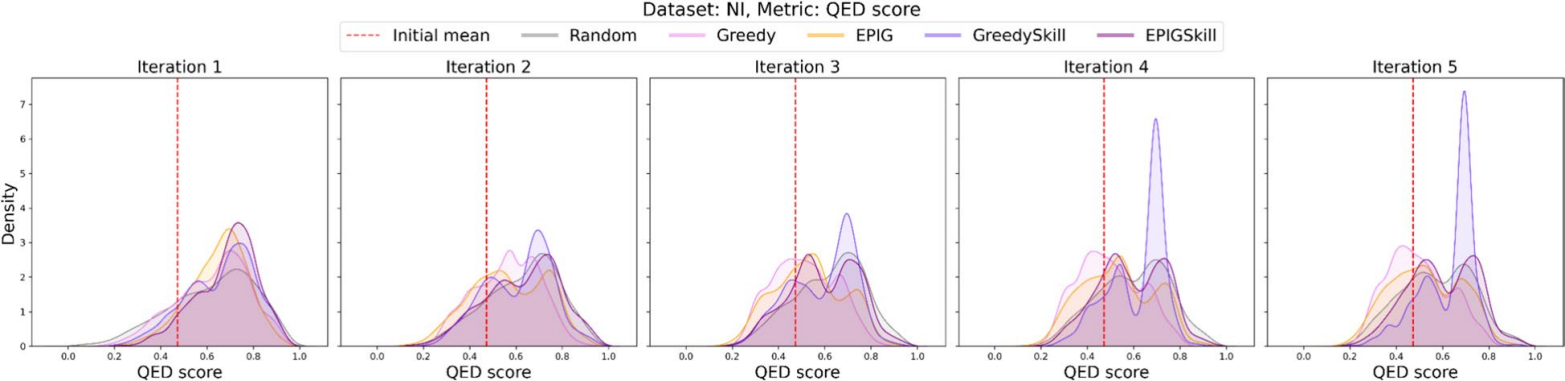
Distributions of QED scores of the putative interfering compounds computed across five E-GuARD iterations for the NI data set. The red dashed, vertical line in each panel corresponds to the mean QED score of the interfering compounds in the initial predictor training set

As E-GuARD iteratively generates compounds, ensuring that the generated structures maintain drug-like properties relevant for early drug discovery is essential. To evaluate this, we computed the QED metric (measuring drug-likeness) for the generated interfering compounds, tracking these metrics for each acquisition function. For the NI data, the QED distributions are shown in Fig. 6 (the results for the remaining data sets are reported in Figure S1). For NI, a significant increase in QED (two-sample t-test: T = 30.94, *P* < 0.001, DF = 6416) was achieved when the GreedySkill acquisition function was applied. Indeed, the initial mean QED value of 0.48 increased up to 0.76 by the end of iteration five.

When using EPIGSkill acquisition, a smaller yet noticeable increase in QED was observed (the QED mean reached 0.63 at iteration five). Other acquisition functions did not show any positive contribution to the QED of the generated molecules. The observed improvement in the drug-likeness of generated molecules with human-preference-based acquisition functions (e.g., GreedySkill, EPIGSkill) highlights the importance of human feedback in keeping the generative model's output relevant to drug discovery. Hence, adopting simulated human experts gives the QSIR model additional opportunities to learn challenging cases.

### Student evolution: central kernel alignment (CKA_rf_) analysis

**Fig. 7:**
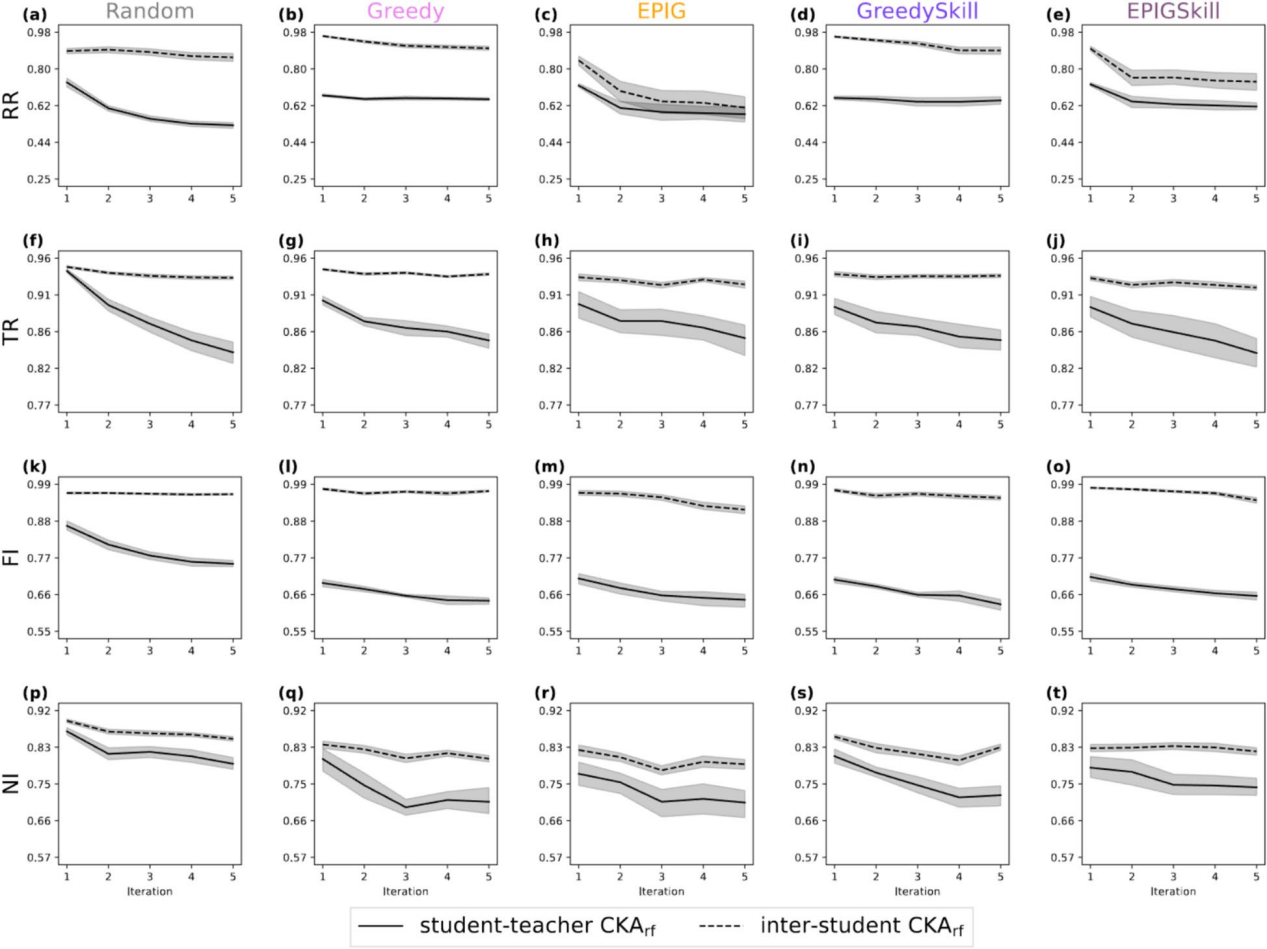
Inter-student CKA_rf_ (dotted lines) measures the similarity between student RFs of different runs, and student–teacher CKA_rf_ (solid lines) measures the similarity between student RFs and teacher RF

Understanding the evolution of classifiers in response to augmented training data is critical for assessing E-GuARD's iterative training effectiveness. To (i) quantify the effect of supplementing the training data of the model with generated molecules and (ii) check for consistency across runs, we used CKA_rf_ to measure similarity between initial teacher and student models and between student models of different runs, respectively. Figure 7 shows the average CKA_rf_ between the initial teacher and the student of the ten runs and the average CKA_rf_ between all pairwise student model combinations of different runs.

As more augmented data is added to the training set throughout the iterations, the similarity between the teacher and student models decreases, especially for Random acquisition, where the difference between the last and first iteration was, on average, − 0.13 across data sets and runs, confirming that supplementing the model with additional data changes how the RFs partition the test data.

Because Random selection favors exploration, the similarity between student and teacher is generally higher for the Random acquisition function than for Greedy and EPIG, especially at earlier iterations. This effect is most notable for the FI data set, where the average CKA_rf_ between the teacher model and the student model at iteration 1 was 0.87 ± 0.02, while all other acquisition functions achieved an average CKA_rf_ of less than 0.72.

The inter-student CKA_rf_ remained above 0.91 across iterations for the FI and TR data set, confirming that the decision rules remained consistent with respect to the test set. Notably, in most experiments, across runs, student models kept a close similarity within each iteration while diverging from the initial teacher model (the dashed line is above the solid line, with the exceptions being panels (c) and (e)). This suggests that the student models evolve similarly regardless of the specific molecule selection in a given run.

### Prediction of interfering compounds

**Fig. 8:**
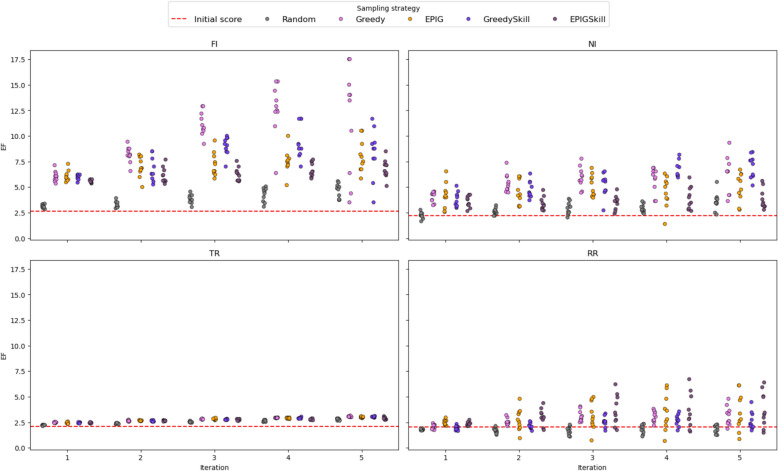
Strip plots displaying the evolution of EF values across five iterations for each data set. The red dashed lines indicate the initial performance computed with the model trained on non-augmented data

We analyzed the evolution of EF and MCC metrics across ten independent runs to evaluate how E-GuARD enhances QSIR model performance over consecutive iterations. As shown in Fig. 8, the baseline models already achieved EF values above 2.0 across all data sets. Successive iterations with E-GuARD-guided augmentation improved these values. Notable gains include an EF increase of 18.0 for FI, 10.0 for NI, and 3.5 for TR when using the Greedy, GreedySkill, and EPIGSkill acquisition functions. The minor improvement observed for TR reflects its more balanced initial data set, leaving less room for augmentation benefits.

The choice of the acquisition function plays a critical role in driving performance improvements. While random sampling consistently underperformed, strategic selection through Greedy, GreedySkill, and EPIGSkill led to the most substantial and consistent gains across data sets. Interestingly, the RR data set showed variable performance, but EF values greater than 6.0 were still achieved for multiple model instances.

**Fig. 9:**
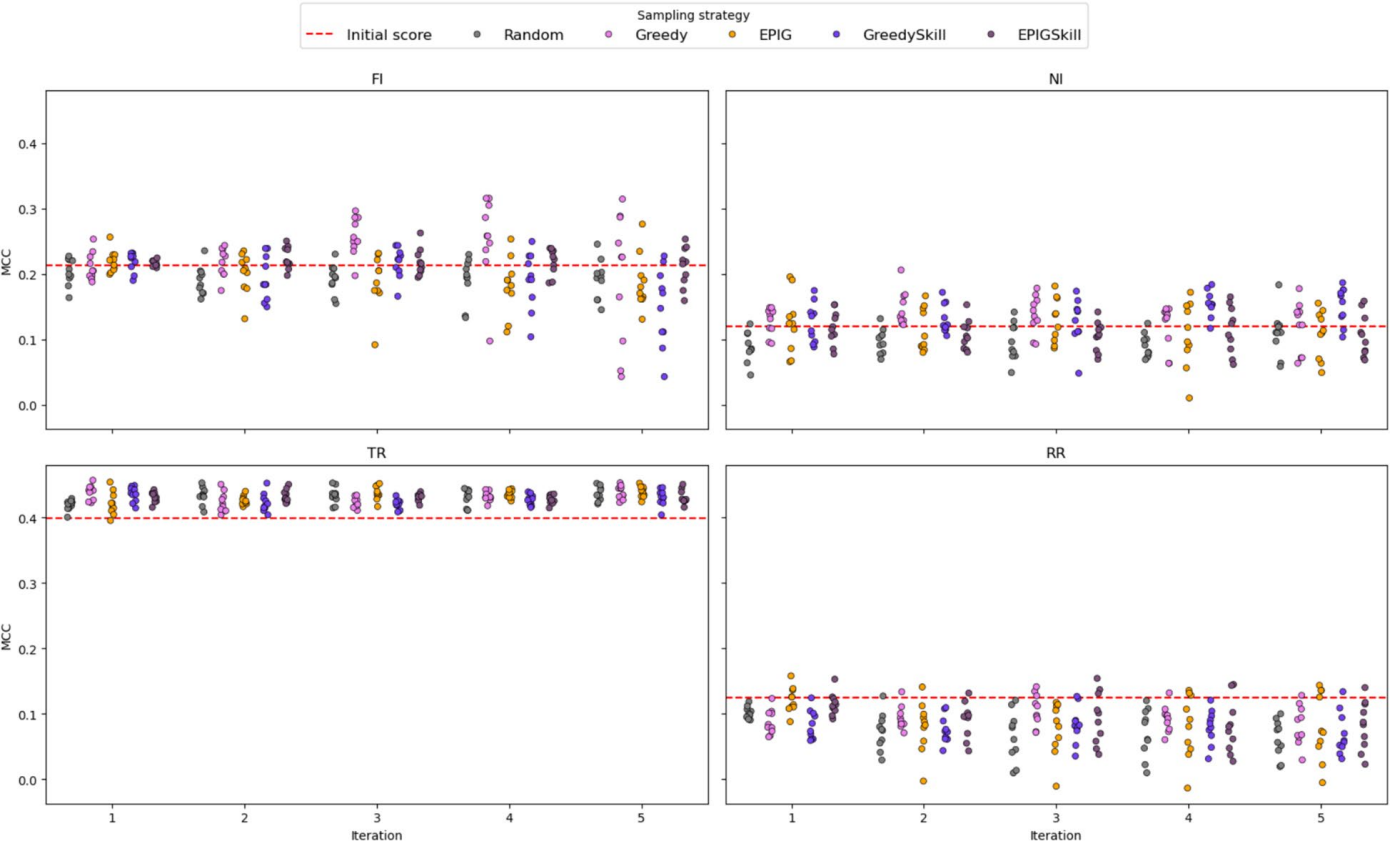
Strip plots displaying the evolution of the MCC score computed across iterations for each data set. The red dashed line indicates the initial performances calculated with the model trained on non-augmented data

Beyond EF, we also evaluated the impact of E-GuARD on overall classification performance using MCC. As shown in Fig. 9, E-GuARD improved MCC scores for three out of four data sets. Notably, data sets with moderately strong baseline models, such as TR, exhibited the largest gains, with MCC rising from 0.39 to a maximum of 0.46. For weaker baseline models, such as FI (initial MCC = 0.22), significant gains (t-test: T: 4.04; *P* = 0.002, DF = 9) were observed with the Greedy acquisition strategy, achieving a peak MCC average of 0.26 at iteration 3.

The results were mixed for data sets with low initial MCC values, such as RR (MCC = 0.12) and NI (MCC = 0.09). In the NI data set, E-GuARD induced specific improvements over initial values in early iterations, reaching a maximum MCC mean of 0.15 with the Greedy (t-test: T = 6.58, P < 0.001, DF = 9) and GreedySkill (t-test: T: 10.04, P < 0.001, DF = 9) acquisition functions. The average MCC across the 10 independent runs for the RR data set did not improve but remained consistent with the initial model, indicating that while positive predictive performance increased, overall classification performance was not compromised. Additional metrics and t-test significance statistics are provided in the Supplementary Materials (Tables S2–S6).

Additionally, we conducted a t-test statistical analysis of the performance of all four selection strategies (Greedy, GreedySkill, EPIG, and EPIGSkill) applied to the four tasks (NI, FI, TR, RR) using different evaluation metrics: MCC, EF, balanced accuracy, and the precision-recall area under the curve (PR AUC). Specifically, we report the results from the E-GuARD iteration that achieved the highest MCC mean value across the 10 independent runs for each acquisition strategy and compare these results to the same iteration under random sampling. The results indicate that the different selection strategies consistently outperformed random data selection across the four tasks and evaluation metrics to varying degrees. EF showed the most consistent improvement across all tasks and strategies, with GreedySkill demonstrating the most substantial impact (NI: T = 13.62, P < 0.001, DF = 13.54; FI: T = 29.21, P < 0.001, DF = 15.97; TR: T = 13.50, P < 0.001, DF = 17.50; RR: T = 1.90, P = 0.08, DF = 10.59). The MCC improved significantly in most cases, particularly with Greedy and EPIGSkill (e.g., Greedy—NI: T = 4.88, P < 0.001, DF = 16.18; FI: T = 5.50, P < 0.001, DF = 16.73; TR: T = 4.51, P < 0.001, DF = 16.43; RR: T = 2.83, P = 0.01, DF = 15.24). Balanced accuracy, however, did not show significant improvements and, in some cases, even declined significantly (e.g., GreedySkill—NI: T = 0.83, P = 0.42, DF = 17.65; FI: T = − 9.86, P < 0.001, DF = 12.87; TR: T = − 2.0, P = 0.06, DF = 14.78; RR: T = − 4.34, P < 0.001, DF = 12.63). The PR AUC improvements were inconsistent, with Greedy and GreedySkill frequently outperforming the random baseline (e.g., Greedy—NI: T = 2.69, P = 0.01, DF = 16.35; FI: T = − 0.99, P = 0.34, DF = 11.81; TR: T = 4.34, P < 0.001, DF = 17.97; RR: T = 3.48, P = 0.002, DF = 16.57), while EPIG and EPIGSkill show mixed results. Overall, Greedy and EPIGSkill provided the most reliable significant improvements across tasks compared to the Random sampling baseline, particularly in EF and MCC, making them the most effective selection strategies.

### External validation on PubChem bioassay data

To further evaluate the impact of the E-GuARD approach on predicting compounds that interfere with biological assays, we conducted additional experiments on an external dataset (AID411) sourced from the PubChem Bioassay database.

**Fig. 10:**
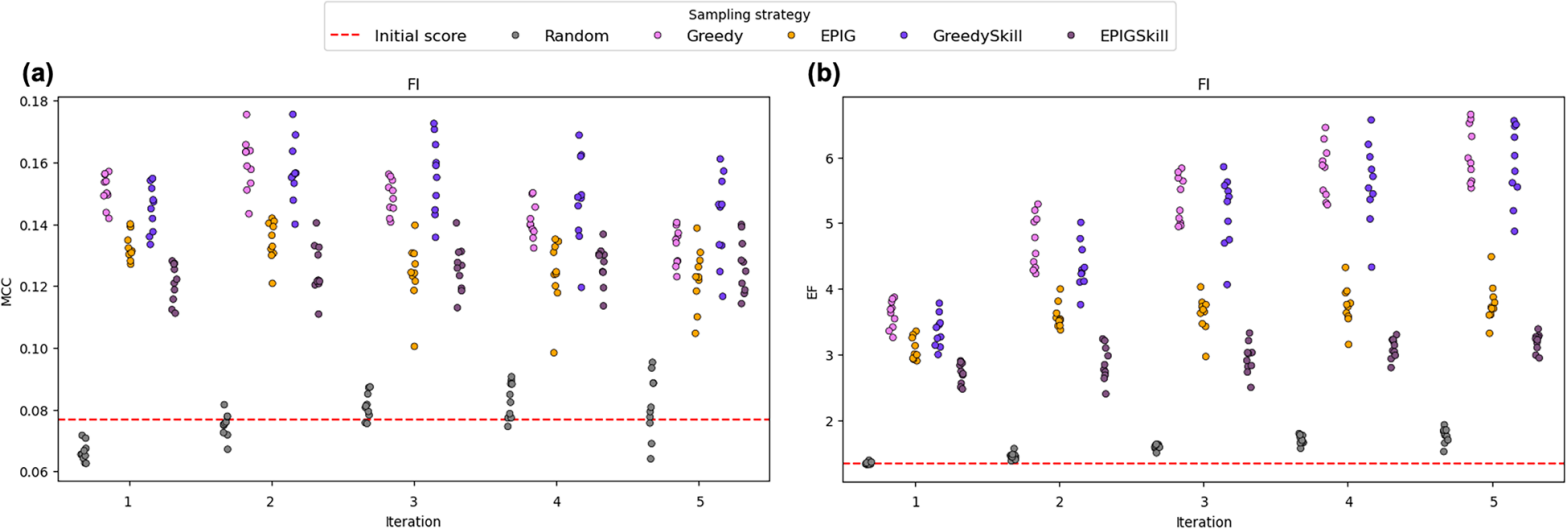
Strip plots illustrating the evolution of (**a**) MCC and (**b**) EF scores throughout E-GuARD iterations on the external dataset. The red dashed line indicates the initial performance of the threshold-optimized baseline model

In this study, we adopted a threshold-optimized version of the FI teacher model to assess whether threshold tuning alone could account for performance gains. However, as shown in Fig. 10, E-GuARD consistently improved model performance beyond what can be achieved through threshold optimization alone, with a maximum observed increase of the MCC by 0.1 and the EF by 6.

The initial low performance of the baseline models likely relates to the external data set extending beyond the chemical space on which the original models were trained, significantly complicating the prediction task. Furthermore, differences in assay conditions between the external data set and the original training set introduce a degree of aleatoric uncertainty, which may further affect model performance.

Despite these challenges, we continue to observe a consistent performance improvement with E-GuARD, highlighting its potential utility in addressing data scarcity and enhancing model generalization.

## Conclusions

This work introduces E-GuARD, a powerful approach to predicting assay interference compounds that integrates self-distillation, active learning, and expert-guided molecular generation. We show that E-GuARD enriches the initial, small training data sets with structurally diverse compounds representing the minority class. The integrated approach enhanced key performance metrics, such as the EF and the MCC, across all four test cases under investigation. Moreover, E-GuARD ensures that data sets remain chemically relevant to drug discovery by integrating human expertise into the data acquisition process.

As our work shows, E-GuARD induces significant performance improvements in machine learning models, which could translate into a smoother hit prioritization process for HTS scientists and medicinal chemists in early drug discovery. By enriching the identification of interference-free compounds, E-GuARD can double the number of true positives compared to standard QSAR models, reducing experimental validation time and costs. For medicinal chemists, E-GuARD offers a cheminformatics-driven method to identify and deprioritize compounds prone to interference, optimizing HTS libraries before synthesis.

However, this work is not without limitations. The data sets used in this study have a limited chemical space and do not cover all possible interference types, highlighting areas for future exploration. While E-GuARD shows great promise, addressing these gaps and expanding its applicability to broader interference types will be key in future research. This pioneering effort lays the groundwork for integrating machine learning with experimental validation to enhance drug discovery efficiency and reliability.

## Supplementary Information

Supplementary material 1.

## Data Availability

The complete data sets used to model assay interference, information on the data set splits employed in this work and all code used to develop and test E-GuARD is available from https://github.com/vincenzo-palmacci/E-GuARD.
