# Supplementary material for: E-GuARD: expert-guided augmentation for the robust detection of compounds interfering with biological assays
Source: J Cheminform. 2025 Apr 29;17:64. doi: 10.1186/s13321-025-01014-3 (PMC12042382; doi:10.1186/s13321-025-01014-3)
Supplement: Supplementary file 1 — Supplementary material 1. [file 13321_2025_1014_MOESM1_ESM.pdf]

SUPPORTING INFORMATION

E-GuARD: Expert-Guided Augmentation for Robust Interference Compound Detection

Vincenzo Palmacci[1,2,#], Yasmine Nahal[3,4,#], Matthias Welsch[1,2,5], Ola Engkvist[4,6], Samuel Kaski[3,7] and Johannes Kirchmair[1,5*]

[1] Department of Pharmaceutical Sciences, Division of Pharmaceutical Chemistry, Faculty of Life Sciences, University of Vienna, 1090 Vienna, Austria

[2] Vienna Doctoral School of Pharmaceutical, Nutritional and Sport Sciences (PhaNuSpo), University of Vienna, 1090 Vienna, Austria

[3] Department of Computer Science, Aalto University, Espoo, Finland

[4] Molecular AI, Discovery Sciences, BioPharmaceuticals R&D, AstraZeneca, Gothenburg, Sweden

[5] Christian Doppler Laboratory for Molecular Informatics in the Biosciences, Department for Pharmaceutical Sciences, University of Vienna, 1090 Vienna, Austria

[6] Department of Computer Science and Engineering, Chalmers University of Technology, Gothenburg, Sweden

[7] Department of Computer Science, University of Manchester, Manchester, United Kingdom

[#] These authors contributed equally to this work.

* Corresponding author: johannes.kirchmair@univie.ac.at

Table S1: Comparison of balanced accuracy between the Liability Predictor and E-GuARD QSIR models for four interference mechanisms using 5-fold cross-validation. For the Liability Predictor, averages, as found in the original publication, are reported. For E-GuARD, both averages and standard deviations are provided.

	**FI**	**NI**	**TR**	**RR**
**Liability predictor**	0.78	0.75	0.70	0.62
**E-GuARD**	0.81 ± 0.01	0.71 ± 0.02	0.73 ± 0.01	0.66 ± 0.02

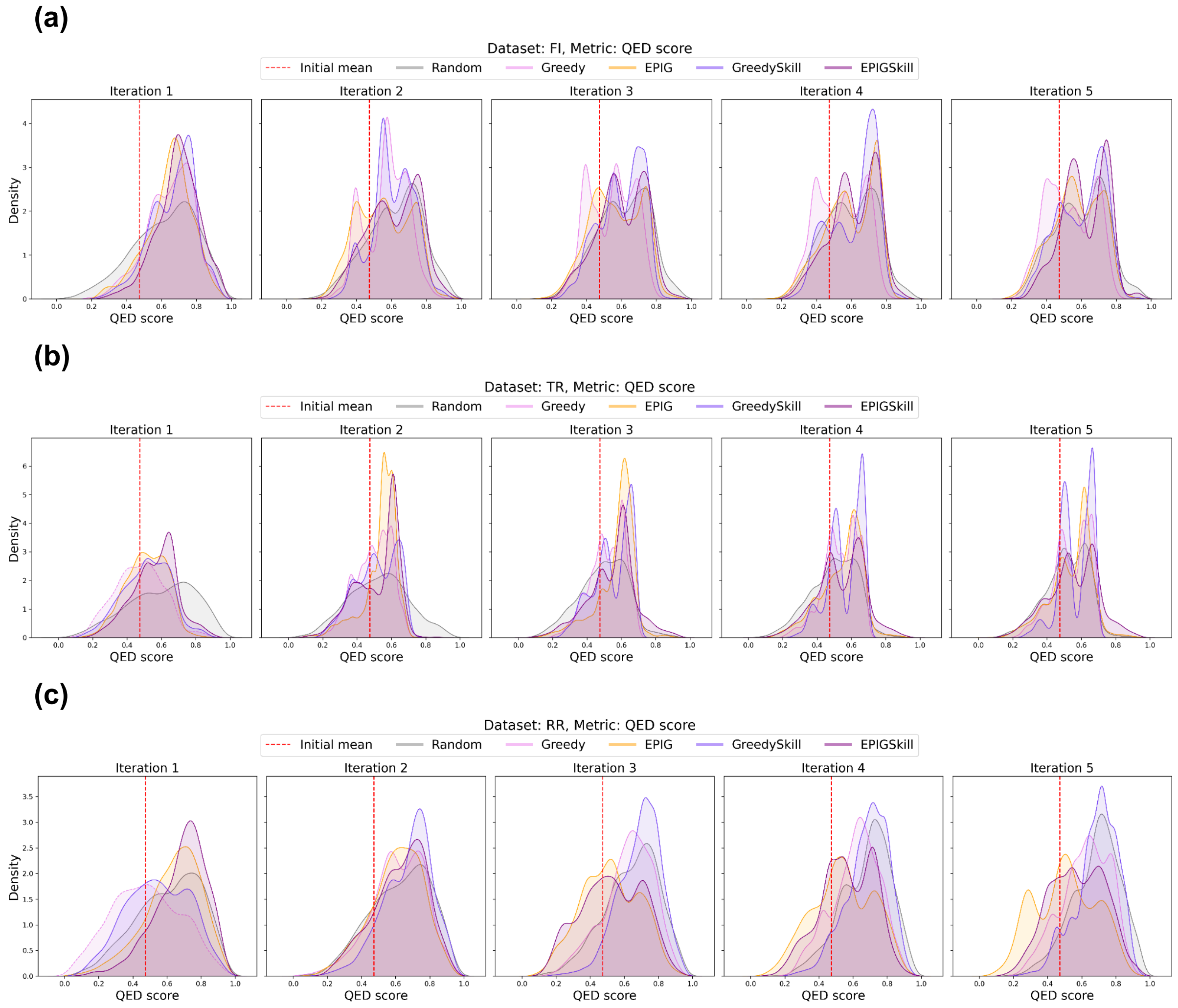

Figure S1: Distributions of QED scores of the putative interfering compounds computed across five iterations of E-GuARD for **(a)** FI, **(b)** TR and **(c)** RR data sets. The red dashed, vertical line in each panel corresponds to the mean QED score of the interfering compounds in the initial predictor training set. Expert-guided acquisition strategies such as GreedySkill (in blue) and EpigSkill (in purple) lead to higher QED scores than other acquisition strategies and the initial mean QED value, indicating that more interfering compounds possessing drug-like properties are being added to the training set throughout the E-GuARD process.

Comparison of metric performances across interference tasks using a specific data selection strategy. A t-test (scipy.stats) is used to compare the initial metric value (representing the teacher model performance at iteration i=0) with values from models updated with E-GuARD from each run (we performed 10 runs in total). Metrics are averaged over the 10 independent runs, with t-test statistics (9 degrees of freedom) reported below each mean. Significant improvements (*P* < .01) are highlighted in bold.

Table S2: Reported mean metric values at the best performing E-GuARD iteration (denoted as i*) when using Random data sampling for each task interference task. i* corresponds to the iteration where the highest MCC value was observed.

Task	MCC (i=0)	MCC (i*)	EF (i=0)	EF (i*)	Balanced Accuracy (i=0)	Balanced Accuracy (i*)	PR AUC (i=0)	PR AUC (i*)
**NI (i*=5)**	0.09	0.11<br>T=1.64<br>*P*=.13	**1.96**	**3.57**<br>T=5.83<br>*P*<.001	0.68	0.64<br>T=-3.39<br>*P*=.007	0.07	0.06<br>T=-2.04<br>*P*=.07
**FI (i*=1)**	0.22	0.2<br>T=-2.43<br>*P*=.03	**2.74**	**3.14**<br>T=7.16<br>*P*<.001	0.82	0.76<br>T=-5.88<br>*P*<.001	0.26	0.17<br>T=-16.25<br>*P*<.001
**TR (i*=5)**	**0.4**	**0.44**<br>T=9.44<br>*P*<.001	**2.17**	**2.78**<br>T=24.38<br>*P*<.001	0.73	0.71<br>T=-20.08<br>*P*<.001	**0.53**	**0.55**<br>T=13.04<br>*P*<.001
**RR (i*=1)**	0.12	0.1<br>T=-4.95<br>*P*<.001	1.92	1.77<br>T=-6.85<br>*P*<.001	0.66	0.64<br>T=-4.31<br>*P*=.001	0.12	0.09<br>T=-4.73<br>*P*=.001

Table S3: Reported mean metric values at the best performing E-GuARD iteration (denoted as i*) when using Greedy data sampling for each task interference task. i* corresponds to the iteration where the highest MCC value was observed.

Task	MCC (i=0)	MCC (i*)	EF (i=0)	EF (i*)	Balanced Accuracy (i=0)	Balanced Accuracy (i*)	PR AUC (i=0)	PR AUC (i*)
**NI (i*=2)**	**0.09**	**0.15**<br>T=6.58<br>*P*<.001	**1.96**	**5.41**<br>T=12.18<br>*P*<.001	0.68	0.65<br>T=-3.90<br>*P*=.003	0.07	0.1<br>T=2.57<br>*P*=.03
**FI (i*=3)**	**0.22**	**0.26**<br>T=4.04<br>*P*=.002	**2.74**	**11.21**<br>T=22.39<br>*P*<.001	0.82	0.61<br>T=-39.11<br>*P*<.001	0.26	0.18<br>T=-20.35<br>*P*<.001
**TR (i*=1)**	**0.4**	**0.44**<br>T=9.77<br>*P*<.001	**2.17**	**2.47**<br>T=19.88<br>*P*<.001	0.73	0.74<br>T=2.17<br>*P*=.05	**0.53**	**0.54**<br>T=10.97<br>*P*<.001
**RR (i*=3)**	0.12	0.11<br>T=-1.22<br>*P*=.25	**1.92**	**3.1**<br>T=6.66<br>*P*<.001	0.66	0.58<br>T=-13.26<br>*P*<.001	0.12	0.07<br>T=-9.21<br>*P*<.001

Table S4: Reported mean metric values at the best performing E-GuARD iteration (denoted as i*) when using GreedySkill data sampling for each task interference task. i* corresponds to the iteration where the highest MCC value was observed.

**Task**	**MCC (i=0)**	**MCC (i*)**	**EF (i=0)**	**EF (i*)**	**Balanced Accuracy (i=0)**	**Balanced Accuracy (i*)**	**PR AUC (i=0)**	**PR AUC (i*)**
**NI (i*=4)**	**0.09**	**0.15** T=10.04 *P*<.001	**1.96**	**6.68** T=19.18 *P*<.001	0.68	0.63 T=-7.58 *P*<.001	0.07	0.08 T=1.86 *P*=.09
**FI (i*=1)**	0.22	0.22 T=-.07 *P*=.94	**2.74**	**5.98** T=40.41 *P*<.001	0.82	0.66 T=-35.31 *P*<.001	0.26	0.21 T=-9.93 *P*<.001
**TR (i*=1)**	**0.4**	**0.44** T=8.49 *P*<.001	**2.17**	**2.45** T=21.26 *P*<.001	0.73	0.74 T=1.34 *P*=.21	**0.53**	**0.54** T=5.38 *P*<.001
**RR (i*=1)**	0.12	0.08 T=-4.52 *P*=.001	1.92	1.91 T=-.05 *P*=.96	0.66	0.6 T=-6.75 *P*<.001	0.12	0.06 T=-39.54 *P*<.001

Table S5: Reported mean metric values at the best performing E-GuARD iteration (denoted as i*) when using EPIG data sampling for each task interference task. i* corresponds to the iteration where the highest MCC value was observed.

**Task**	**MCC (i=0)**	**MCC (i*)**	**EF (i=0)**	**EF (i*)**	**Balanced Accuracy (i=0)**	**Balanced Accuracy (i*)**	**PR AUC (i=0)**	**PR AUC (i*)**
**NI (i*=3)**	**0.09**	**0.13** T=3.46 *P*=.007	**1.96**	**5.17** T=9.61 *P*<.001	0.68	0.63 T=-4.50 *P*=.001	0.07	0.07 T=.12 *P*=.90
**FI (i*=1)**	0.22	0.22 T=-.08 *P*=.93	**2.74**	**6.07** T=19.86 *P*<.001	0.82	0.66 T=-45.74 *P*<.001	0.26	0.21 T=-4.77 *P*=.001
**TR (i*=5)**	**0.4**	**0.44** T=12.89 *P*<.001	**2.17**	**3.01** T=46.27 *P*<.001	0.73	0.7 T=-26.20 *P*<.001	**0.53**	**0.55** T=15.58 *P*<.001
**RR (i*=1)**	0.12	0.12 T=.98 *P*=.35	**1.92**	**2.48** T=7.37 *P*<.001	0.66	0.64 T=-3.19 *P*=.01	0.12	0.09 T=-5.02 *P*<.001

Table S6: Reported mean metric values at the best performing E-GuARD iteration (denoted as i*) when using EPIGSkill sampling for each task interference task. i* corresponds to the iteration where the highest MCC value was observed.

Task	MCC (i=0)	MCC (i*)	EF (i=0)	EF (i*)	Balanced Accuracy (i=0)	Balanced Accuracy (i*)	PR AUC (i=0)	PR AUC (i*)
**NI (i*=1)**	**0.09**	**0.12** T=3.25 *P*=.009	**1.96**	**3.57** T=9.10 *P*<.001	0.68	0.66 T=-2.14 *P*=.06	0.07	0.06 T=-.86 *P*=.41
**FI (i*=2)**	0.22	0.22 T=.89 *P*=.39	**2.74**	**6.14** T=14.14 *P*<.001	0.82	0.66 T=-28.62 *P*<.001	0.26	0.18 T=-10.05 *P*<.001
**TR (i*=2)**	**0.4**	**0.43** T=9.65 *P*<.001	**2.17**	**2.64** T=36.27 *P*<.001	0.73	0.72 T=-7.23 *P*<.001	**0.53**	**0.54** T=8.72 *P*<.001
**RR (i*=1)**	0.12	0.12 T=-.10 *P*=.92	**1.92**	**2.33** T=7.09 *P*<.001	0.66	0.63 T=-3.75 *P*=.004	0.12	0.09 T=-6.35 *P*<.001
